# Outpatient multimodal intravenous analgesia in patients undergoing day-case surgery: description of a three year experience

**DOI:** 10.1186/s12871-016-0246-6

**Published:** 2016-09-13

**Authors:** Magdalena Serra, Roser Vives, Montserrat Cañellas, Josep Planell, Joan Carles Oliva, Carmen Colilles, Caridad Pontes

**Affiliations:** 1 Anesthesiology Department, Hospital de Sabadell, Institut Universitari Parc Taulí – Universitat Autònoma de Barcelona, Sabadell, Barcelona Spain; 2 Clinical Pharmacology Unit, Hospital de Sabadell, Institut Universitari Parc Taulí – Universitat Autònoma de Barcelona, Sabadell, Barcelona Spain; 3 Statistics Unit, Institut d’Investigació e Innovació Parc Taulí, Sabadell, Barcelona Spain; 4 Departamento de Farmacologia, de Terapèutica i de Toxicologia, Universitat Autònoma de Barcelona, Edifici Taulí planta −1 Hospital de Sabadell C/ Parc taulí n° 1, Sabadell, Barcelona 08208 Spain

**Keywords:** Domiciliary care, Ambulatory Surgery, Elastomer, Intravenous Analgesia

## Abstract

**Background:**

The use of elastomeric devices for ambulatory intravenous pain treatment in Major Ambulatory Surgery (MAS) has been described to improve postoperative pain management. The objective of the study was to describe the first 3 years experience of the use of elastomeric devices for ambulatory intravenous pain treatment in MAS implemented at our site since 2010.

**Methods:**

Data were retrieved from the medical records for all patients who, between January 2010 and March 2014, underwent surgical procedures at the ambulatory surgical centre at our hospital and were prescribed a home-based continuous intravenous analgesia.

**Results:**

Data were retrieved from the medical records of 1128 patients. The most frequent surgical interventions included orthopedic and proctology surgeries. 80 % of patients were discharged home without pain; during the first 48 h after discharge roughly 40 % of subjects were completely free of pain, 50 % reported mild pain (VAS 1 to 3) and 9 % reported higher pain scores (4 and above). Peripheral nerve block was associated to better pain control in the immediate postoperative period. Vomiting in the first 24 h was 4.6 % before introducing haloperidol into the drug schemes, and 2.6 % thereafter. Complications related with the intravenous route required treatment withdrawal in 1.1 % cases. Only 3.5 % of patients returned to the hospital in the first 72 h, mainly for non-pain related reasons. Overall, 99.5 % of patients were satisfied with the treatment received at home.

**Conclusion:**

Our initial experience suggest that outpatient multimodal intravenous analgesia in patients undergoing day-case surgery is a feasible alternative in our setting, that allows an effective management of postoperative pain with a small rate of adverse events and complications requiring readmission.

## Background

Acute postoperative pain (APP) and postoperative nausea and vomiting are the most frequent clinical problems after Major Ambulatory Surgery (MAS). They complicate the recovery, delaying patient discharge or increasing the rate of readmission after discharge. The development of new minimally invasive surgical techniques, the progress in anesthesia and new systems for administration of analgesics have been instrumental to expand the scope of surgical procedures that can be done in MAS settings, including interventions which were previously discarded mainly due to the difficulties to achieve an appropriate control of APP [1, 2].

An appropriate control of pain is a healthcare quality indicator having social and economical implications. However, in US and Canada, where about 70 % of surgical procedures are MAS, 30 % of subjects undergoing ambulatory surgery yet suffer moderate to severe pain in the first 24 h after surgery, despite receiving analgesic treatment [3]. Thus, there is a need to do a careful selection of those procedures and the patients which are appropriate for ambulatory surgery in each specialty [4].

There is general agreement on multimodal analgesia as the most appropriate analgesia in MAS [1, 5–9]. In our setting, the ASECMA (*Asociación Española de Cirugía Mayor Ambulatoria-* Spanish Association for Major Ambulatory Surgery) subscribed a joint consensus document with other medical-surgical societies, which includes a statement on the need to apply a multidisciplinary, multimodal and individualized approach to surgical pain management [10].

Post-operative pain in MAS should be approached already before surgery, with home preoperative medication, and consolidated in the intra-operative period with appropriate anesthesia and analgesia; in particular, nerve blockades, infiltrations and/or instillation of surgical wounds with long lasting local anesthetics have proven to be very effective and should be done systematically [3]. In the postoperative period, pain management is based on intravenous analgesia while still in the ambulatory surgery unit (ASU), followed by ambulatory analgesia at patient’s home [11–13]. While oral analgesia at home obtains good results when mild to moderate pain is expected, some studies have shown that it has high rates of failure in moderate to severe pain, with up to 7 % of patients requiring hospitalization and intravenous treatment for uncontrolled postoperative pain [14, 15].

The availability of elastomeric infusion systems and pumps for intravenous administration both as continuous infusion and as patient controlled analgesia (PCA) allow stable plasma concentrations of drugs which achieve better control of pain, thus improving efficacy and reducing adverse effects as compared to bolus administration [16, 17].

At our site and in parallel to an early discharge program in MAS, we introduced the use of elastomeric devices for early treatment of postoperative pain in MAS patients on 2008, with the aim to improve postoperative pain management in the first days after surgery. On January 2010 a formal protocol for analgesia in postoperative pain in patients undergoing ambulatory surgery was issued and implemented, which included the use of elastomeric pumps for intravenous analgesia before and after discharge, covering the first 48 to 96 h after surgery depending on the degree of expected pain for each type of surgery. The protocol includes close monitoring by nursing staff for as long as the patient is receiving intravenous treatment.

The present report summarizes the three year experience with the protocol for domiciliary intravenous analgesia for the treatment of postoperative pain after major ambulatory surgery, with special emphasis on describing the pain control achieved, and with the aim to identify any specific safety problems that may arise from the application of domiciliary analgesia.

### Methods

#### Design

An early discharge program in MAS with nurse-driven ambulatory follow up was implemented on January 2010. As a part of pain management, the protocol included the use of elastomeric pumps for continuous intravenous analgesia administration during the first 48 to 96 h post-surgery. In February 2014, an observational retrospective study was designed aimed to describe the initial experience regarding the effectiveness and safety of the use of elastomeric pumps for analgesia in this setting. The study protocol was reviewed and approved by an independent ethic’s committee (CEIC Corporació Sanitaria Parc Taulí, Sabadell) on March 4^th^ 2014; the requirement for written informed consent was waived by the institutional review board.

#### Patients

Data were retrieved from the medical and nursing records for all patients who, between January 2010 and March 2014, underwent surgical procedures in the CQA (ambulatory surgical centre, Hospital de Sabadell, Barcelona) and were prescribed a home-based continuous intravenous analgesia (HBCIA); a semi-structured questionnaire was used for patient follow-up as a part of the protocol activities. Table 1 summarizes the type of surgeries that the protocol established as potential candidates and the exclusion criteria for home-based iv analgesia.

**Table 1:** Criteria for home based intravenous analgesia following major ambulatory surgery

Criteria for home-based continuous intravenous analgesia (HBCIA) after ambulatory surgery at our centre
− Type of surgeries eligible for home based iv analgesia − Shoulder surgeries: acromioplasty and other shoulder arthroscopic procedures − Upper limbs surgeries: arthroplasty, rhizarthrosis surgery, osteotomy, osteosynthesis − Lower limb surgeries: knee ligamentoplasty, hallux valgus surgery, foot osteotomy, foot arthroplasty − Proctologic surgery − Any surgery anticipated to induce moderate or intense pain.
Patients not candidates for invasive home analgesia
− Patients with cognitive dysfunction − History of drug abuse or psychiatric disease − Patients without family support at home − Patients with known drug allergies − Patients that live within a distance from the hospital considered too far that would make the visit of the nurses not feasible

#### Treatments

During this period, five different analgesic schemes were used for HBCIA (Table 2). The choice of scheme for a given patient was decided by the anesthesiologist according to the patient’s characteristics (type of surgery, age, weight and safety considerations).

**Table 2:** Description of the different elastomeric pumps

	*N*	%
Description		
Tramadol 400 mg + dexketoprofen 250 mg + haloperidol 2,5 mg in physiological saline (total volume 100 cc), 2 ml/h, 48 h	912	80.1 %
Tramadol 400 mg + dexketoprofen 250 mg in physiological saline (total volume 100 cc), 2 ml/h, 48 h	154	13.6 %
Tramadol 200 mg + dexketoprofen 100 mg in physiological saline (total volume 50 cc), 2 ml/h, 24 h	40	3.5 %
Tramadol 300 mg + 2.5 mg haloperidol in physiological saline (total volume 100 cc), 2 ml/h, 48 h Ibuprofen 600 mg/8 h oral route	12	1.1 %
Tramadol 400 mg + metamizole 2 g in physiological saline (total volume 100 cc), 2 ml/h, 48 h	5	0.44 %
Not registered	5	0.44 %
Total	1128	

All patients were prescribed Paracetamol 1 g/6 h oral route, gastric protection and metoclopramide 10 mg/12 oral route if needed

The elastomeric pumps used were Infusor Folfusor SV 2 (Baxter) which is a portable and disposable non-electronic small volume infuser with nominal flow rate of 2 mL/h and a maximum volume of 130 mL, which allows a slow continuous infusion over 48 h. The pumps were prepared by the hospital Pharmacy Department and delivered to the CQA, where nursing personnel could add to the pump customized antiemetic medications according to the postoperative medical prescriptions.

Infusions using HBCIA elastomeric pumps were started either at the anesthetic reanimation room or at the so called unit for adaption to environment, and were maintained for 48 h, regardless of patient discharge to their home. Patients were instructed on general care of venous access and pump care. After 24 h patients received a follow-up call by a nurse, and at 48 h they received a nurse visit at home; in some cases, a new pump was replaced during this visit to prolong HBCIA for additional 24 h or even 48 h (mainly limited to hemorrhoidectomies), as prescribed depending on the intensity of pain. At the end of the prescribed period (48 h, 72 h or 96 h) the nurse retired the pump and iv access, in addition to reviewing of wounds and dressings, and checked pain and wellbeing. At any time, nurses solved problems derived from the infusion (extravasation, dislocation of the route), or stopped the infusion in case of adverse reactions (nausea, phlebitis…) and derived to the hospital any problem related with the infusion, the drugs infused or any postoperative complication which required medical assessment.

#### Outcomes and measurements

Medical and nursing records were reviewed to retrieve demographic data, surgical and anesthetic procedures, and treatments administered before hospital discharge and during the post-surgery period.

The main effectiveness assessments were numerical rating scales (0–10) for post-operative pain which were routinely assessed by the semi-structured form used by nurses for patient follow-up, as a part of the protocol activities, at entry and discharge of each care unit, and also once daily during ambulatory care.

Also, safety information was obtained from the routine assessments performed before discharge and after during ambulatory visits. The semi-structured forms included assessments for presence of nausea, vomiting, somnolence and pruritus, need for analgesic rescue medication, incidences with the infusion system and patient satisfaction (satisfied or not satisfied). Any further hospital admissions or visits to emergency ward were retrieved from the hospital admission records.

#### Statistical methods

Quantitative variables were described and are presented as mean ± standard deviation, and qualitative variables with number and percentages, considering the different transitions of care from reanimation to the unit for adaption to environment, and then daily for the home care period, either on the phone or during a home visit. The numerical pain rating scale was categorized as no pain (0), mild pain (1–3), moderate pain (4–6) and intense pain (7–10).

## Results

From January 2010 to March 2014, 1135 patients underwent major outpatient surgery at CQA who were prescribed home-based analgesia with an elastomeric pump. Of these, 5 patients did not finally receive treatment with elastomeric pumps: 4 because they were admitted to regular hospitalization due to nausea, sickness or pain, during the immediate postoperative period, and 1 due to technical problems with the pump which precluded its use. Two more patients were excluded from description because no nursing visits were done in the first two days due to logistic problems (snow storm). Table 3 describes the characteristics of the remaining 1128 patients. The majority of patients underwent orthopedic surgeries. Throughout the years the number of treated patients per year increased from 216 in 2010 to 299 in 2013, paralleling increases in MAS activity at our site.

**Table 3:** Description of the studied population

	*N*	%
Gender		
Male	467	41.4 %
Females	661	58.6 %
ASA		
ASA 1	370	32.9
ASA 2	668	59.2
ASA 3	70	6.2
Not registered	20	1.8
Type of surgery		
Foot	431	38.2 %
Hand	148	13.1 %
Knee	157	13.9 %
Shoulder	210	18.6 %
Ano-rectal	123	10.9 %
Others	59	5.2 %
	Mean (SD)	range
Age (years)	49.60 (16.01)	15 - 87
Total	1128	

Table 4 describes the type of anesthesia performed. Most patients (67.5 %) received a peripheral nerve block, either as a single anesthetic technique or on top of general or intradural anesthesia. Pre-emptive analgesia with NSAIDs was administered to 71 % for patients undergoing shoulder surgery, but only to 16.9 % in foot interventions. Antiemetic prophylaxis with a single preoperative dose of dexamethasone was given to 78.5 % of patients.

**Table 4:** Type of anesthesia and premedication with NSAIDs and dexamethasone

	*N*	%
Type of anesthesia		
General Anesthesia	440	39.0
*+ peripheral nerve block*	*255*	*22.6*
Intradural anesthesia	186	16.5
*+ peripheral nerve block*	*9*	*0.8*
Peripheral nerve block only	497	44.1
Sedation ± local infiltration	5	0.4
Total	1128	100 %
Any peripheral nerve block		
Yes	761	67.5 %
No	367	32.5 %
Total	1128	100 %
Type of peripheral nerve block		
Suprascapular nerve block	219	28.8 %
Supraclavicular nerve block	40	5.3 %
Foot nerve block	412	54.1 %
Axillary nerve block	85	11.2 %
Other (popliteal, femoral, interscalene)	5	0.7 %
Total	761	100 %
Premedication		
Dexamethasone	886	78.5 %
NSAIDs	410	36.3 %

The type of multimodal analgesia used in the elastomeric pumps is described in Table 2. Until November 2010 haloperidol was rarely prescribed (8.8 %). At that time an interim review of data suggested that nausea and vomiting were frequent, so it was decided to systematically consider adding an antiemetic prophylaxis with haloperidol unless contraindicated. Since that date, 94.5 % of patients received treatment regimens containing haloperidol.

**Fig. 1:**
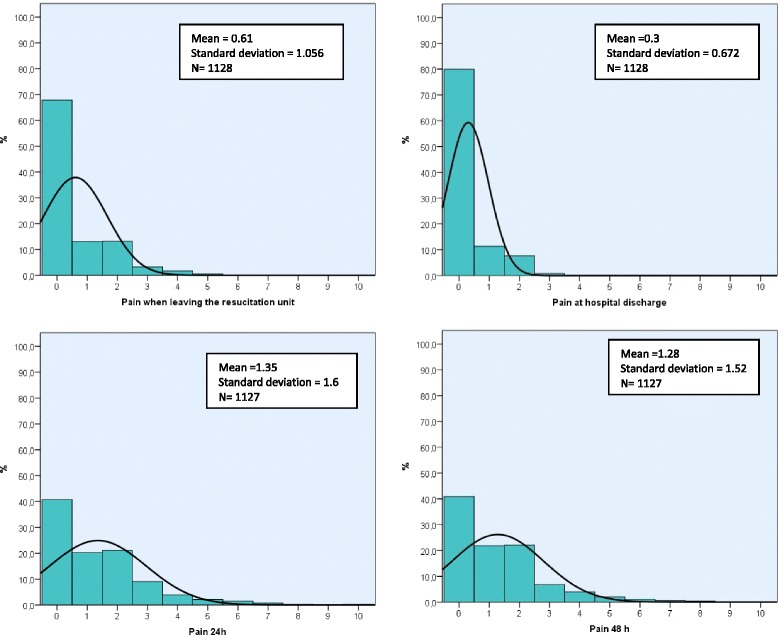
Visual analogue scale results for pain at different times

According to the routine pain assessments, most patients were free of pain immediately after surgery, and only 11.3 % presented moderate or intense pain (pain scores ≥ 4). As expected, knee and shoulder surgery were the most painful interventions with 22.3 % and 25.2 % of patients presenting at least moderate pain, respectively. Nearly 80 % of patients were discharged home without pain, and around 40 % referred to be completely free of pain at home after 24 and 48 h from surgery (Fig. 1). Patients who had undergone hemorrhoid surgery were prescribed longer schedules up to 72–96 h, and often showed peak pain values appearing at 72–96 h (data not shown in the figure).

Overall, patients who had received a peripheral nerve block had better control of pain during the immediate postoperative period, while still at the hospital, as compared to patients without nerve block (% of patients without pain 72.5 % and 48.5 % respectively). 15.4 % of patients used rescue medication with oral tramadol at some time on top of their HBICA.

**Fig. 2:**
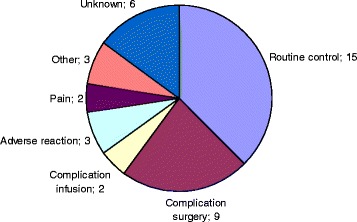
Reasons for post-discharge (within 72 h) patients visits to the hospital

Adverse reactions were infrequent. Intravenous treatment was retired in 5 cases due to emesis and in 8 cases due to other adverse reactions (3 allergic reactions, 3 cases of unspecified intolerance to drugs or infusion pump and 2 arterial thromboses). Complications related with the intravenous route included extravasation in 15 (1.3 %) patients and phlebitis in 4 patients. In 12 (1.1 %) cases the route had to be replaced or retired due to technical problems. During the first 72 h after surgery, 40 patients (3.5 %) returned to the hospital. Figure 2 shows the reasons that were coded in the hospital admission records. Most cases (15) consisted on programmed visits related with the control of the surgical procedure. Table 5 shows the percentage of patients reporting nausea and vomiting during hospital stay, and nausea, vomiting, somnolence and pruritus when called or visited by nurses at home. The incidence of vomiting at 24 h was generally higher in women, and nearly halved after antiemetics were systematically considered as a part of the schedule, since November 2010 (from 4.6 % to 2.6 %). Pre-treatment with dexamethasone, which was given at the anesthesiologist criteria was associated to a vomiting incidence of 2.3 % as compared to 5.8 % when not given. Overall, 99.5 % of patients were satisfied with the treatment received at home.

**Table 5:** Adverse reactions reported

	*N*	%
Nausea and vomiting – surgical wards		
Post-anesthetic resuscitation unit	23	2 %
*Male*	*13*	*2.9 %*
*Female*	*10*	*1.5 %*
Adaptation to media unit	4	0.4 %
*Male*	*2*	*0.4 %*
*Female*	*2*	*0.3 %*
Nausea and vomiting 24 h		
Nausea	60	5.3 %
*Male*	*10*	*2.1 %*
*Female*	*50*	*7.6%* ^a^
Vomiting	34	3 %
*Male*	*3*	*0.6 %*
*Female*	*31*	*4.7%* ^a^
Nausea and vomiting 48 h		
Nausea	61	5.4 %
*Male*	*12*	*2.6 %*
*Female*	*49*	*7.4%* ^a^
Vomiting	33	2.9 %
*Male*	*4*	*0.9 %*
*Female*	*29*	*4.4%* ^a^
Somnolence		
24 h	112	9.9 %
48 h	143	12.7 %
Pruritus		
24 h	13	1.2 %
48 h	21	1.9 %
Patients with at least 1 event	318	28.2 %
Total	1128	

^a^ *P <* 0.01 for Chi-squared comparisons between males and females

## Discussion

Our series describes the experience of more than 3 years with an early discharge program in MAS, in which we introduced the use of elastomeric devices for early treatment of postoperative pain that was extended after discharge, including home-based continuous intravenous analgesia.

In our 1128 patient series, we have observed that pain control while at the surgical centre allowed that 80 % of patients were discharged home with no pain, and that during the first 48 h after discharge, roughly 90 % of subjects were either completely free of pain or reported mild pain scores. However, yet a 9 % reported pain scores of 4 and above.

Most patients received a 48 h infusion and then were switched to oral route; for these, no further pain assessments were available since they were considered completed as regards to special follow-up. Only patients undergoing surgeries considered to be very painful received extended infusions and were followed over 72 or even 96 h; this may be the reason why our data show worse pain control rates at 72 h, with a proportion of patients with scores ≥ 4 up to 14 %.

We consider that our results may be regarded as good, as compared to similar series in both inpatient and outpatient setting. In a case series from the Netherlands, moderate to severe postoperative pain was present in 41 % of hospitalized patients on the first day of surgery, and in 15 % of patients on the 4th day [18]. In the outpatient setting after day-case surgery, on the first 24 h after hospital discharge, Pavlin et al. reported that, up to 60 % of patients reported pain > 4 and up to 20 % pain > 7 [19], and McHugh et al. reported 30 % of patients reported moderate to severe pain, in both cases measured with a verbal rating scale administered during a telephonic survey [20]. Gramke et al. reported that 21 %, 13 % and 10 % of patients had pain VAS > 4 on postoperative days 1, 2, and 3, respectively [21]. To note, most of these series, like ours, include a number of different procedures, mainly orthopedic surgery, but also ear-nose and throat, abdominal, gynecological and breast surgery. Gramke suggests that in his series a number of procedures (head and neck, orthopedic, abdominal and breast plastic surgery) were associated with higher pain [21]. We observed that knee and shoulder surgery were associated with higher pain, but better control of early pain when peripheral nerve block was used during surgery. Thus, it seems reasonable to promote the use of peripheral nerve blocks whenever indicated in these patients. Also, hemorrhoidal surgery was associated with longer-lasting pain, peaking at 72 h, thus justifying the extension of HBCIA up to 96 h in this particular procedure.

All the therapeutic schemes in the elastomeric pumps that were used during the studied period contained tramadol, and almost all (> 97 %) combined both tramadol and dexketoprofen. Routine use of tramadol has been controversial, since nausea and vomiting have been described in up to 40 % of treated patients in some series [22, 23]. Rawal et al. reported that use of oral tramadol after day-case surgery was associated to nausea and dizziness in up to 17.5 %, leading to withdrawal in several patients, and was also associated to patient dissatisfaction [24]. Our experience suggests that tramadol is well tolerated when administered as a part of multimodal analgesia by continuous perfusion, and that tolerability may be improved by adding an antiemetic to the elastomeric pumps. Actually, in our series only 5/1135 (< 0.5 %) patients required treatment withdrawal due to vomiting.

Regarding other clinical outcomes, it is remarkable that we observed a low incidence of complications related to the intravenous route, like extravasation or phlebitis, and the need to replace the venous access or to withdraw the intravenous treatment occurred only in 1.1 % of cases [25]. Also, it is relevant that the main reasons by which patients returned to the hospital in the first 72 h after surgery were not related to pain.

One of the key aspects of our outpatient program is the domiciliary patient follow-up, combining telephonic and in-person nursing care in the early postoperative period. The program uptake was remarkably well accepted by medical and nursing staff [26], but also patients indicated a high rate of satisfaction in our series.

There are a number of limitations to our report. First, this is a report of our clinical experience. Data has been collected retrospectively from clinical charts and simple forms that were designed for clinical purposes during the ambulatory follow at the time of program implementation, and thus the information has not been collected in a standardized and prospective way intended for research purposes. Thus the results reported should be considered within this observational perspective, especially as regards to effectiveness. Also, it has been previously reported that several risk factors can be identified to predict the requirement of prolonged stay in ambulatory surgery [27]. Since the identification of risk factors was not amongst our objectives, in the present study we did not collect a wide description of medical antecedents and conditions of our patients. In the future we may describe different patient profiles with differential requirements, as we increase our experience and collect additional information on small subgroups with higher risk of poor pain control, adverse events or requirements of close medical care.

We present aggregated data for many different surgeries that are known to have quite different pain management requirements and characteristics, and report joint results for a number of different drug preparations. Besides, the type of drug combinations used along the program implementation have been slightly modified in time, so that i. ex. antiemetic drugs, mainly haloperidol, were added to the elastomeric preparations after first year. However, the availability of a number of combinations allowed personalized choices according to each subject characteristics, considering allergies, comorbidities, risks for adverse events and type of surgery. Likely, a reasonable degree of flexibility may be useful to maintain a low rate of complications and improve clinical results.

## Conclusions

Our data suggest that an approach based on extending the intravenous multimodal analgesia after discharge has proven to be feasible and appropriate in a wide series of patients in our setting, and in many surgical procedures, and has proven safe and easy to apply within the parameters defined by our program. Whether this approach may be translated to other settings is uncertain, since the availability of resources, the relatively close proximity of the geographical area to cover, and the easiness of arrangements for home visits after discharge may be difficult to reproduce in other geographies and health system models. We have observed an effective management of postoperative pain with an acceptable rate of adverse events and complications, and the process has been feasible and well accepted by all involved parties. We think that our experience may be useful to other groups who may be considering strategies aimed to enhance patient access to surgical procedures, and since the objectives of the program have been achieved, these results reinforce its continuation and suggest the convenience to consider the extension of the model to other types of surgeries.

## Abbreviations

APP: Acute postoperative pain
ASA: American society of anesthesiology
ASECMA: Asociación Española de Cirugía Mayor Ambulatoria (spanish association for major ambulatory surgery)
ASU: Ambulatory surgery unit
cc: Cubic centimeter
CEIC: Comité ético de investigación clínica (clinical research ethics committee)
CQA: Centre quirúrgic ambulatori (ambulatory surgical center)
g: Gram
h: Hour
HBCIA: Home-based continuous intravenous analgesia
MAS: Major ambulatory surgery
mg: Milligrams
mL: Milliliter
N: Number of patients
NSAID: Non steroidal anti-inflammatory drugs
PCA: Patient controlled analgesia
SD: Standard deviation
VAS: Visual analogue scale
